# Namodenoson at the Crossroad of Metabolic Dysfunction-Associated Steatohepatitis and Hepatocellular Carcinoma

**DOI:** 10.3390/biomedicines12040848

**Published:** 2024-04-11

**Authors:** Ohad Etzion, Avital Bareket-Samish, David Yardeni, Pnina Fishman

**Affiliations:** 1 Department of Gastroenterology and Liver Diseases, Sorkoa University Medical Center, Beer Sheva 84101, Israel; ohadet34@yahoo.com (O.E.); yardeda@gmail.com (D.Y.); 2 BioInsight, Ltd., Binyamina 3056814, Israel; avital@bioinsight-medcom.com; 3 Can-Fite BioPharma, Petah Tikva 49170, Israel

**Keywords:** A3AR, agonist, cirrhosis, clinical trial, fibrosis, liver cancer, hepatocellular carcinoma, namodenoson, metabolic dysfunction-associated steatotic liver disease, metabolic dysfunction-associated steatohepatitis, non-alcoholic fatty liver disease, non-alcoholic steatohepatitis

## Abstract

Namodenoson (CF102) is a small, orally available, anti-inflammatory, and anti-cancer drug candidate currently in phase 2B trial for the treatment of metabolic dysfunction-associated steatohepatitis (MASH; formerly known as non-alcoholic steatohepatitis (NASH)) and in phase 3 pivotal clinical trial for the treatment of hepatocellular carcinoma (HCC). In both MASH and HCC, the mechanism-of-action of namodenoson involves targeting the A3 adenosine receptor (A3AR), resulting in deregulation of downstream signaling pathways and leading to inhibition of inflammatory cytokines (TNF-α, IL-1, IL-6, and IL-8) and stimulation of positive cytokines (G-CSF and adiponectin). Subsequently, inhibition of liver inflammation, steatosis, and fibrosis were documented in MASH experimental models, and inhibition of HCC growth was observed in vitro, in vivo, and in clinical studies. This review discusses the evidence related to the multifaceted mechanism of action of namodenoson, and how this mechanism is reflected in the available clinical data in MASH and HCC.

## 1. Introduction

Adenosine is a ubiquitous endogenous purine nucleoside that is present in most cell types. It is involved in basic biologic functions such as nucleotide biosynthesis and cellular energy metabolism and is an important physiological regulator in many organ systems such as the cardiovascular system, the neurological system, and the immune system. Interestingly, besides the well-known roles of adenosine in normal human physiology, it has been shown to accumulate in the environment of cancer and inflammatory conditions [1]. Adenosine exerts its regulatory role through binding to four adenosine receptors (ARs) that belong to a superfamily of G protein-coupled receptors. The four ARs are A1AR, A2AAR, A2BAR, and A3AR, and they display similar structures (a seven transmembrane-helical structure with an extracellular amino-terminus and an intracellular carboxy terminus) [1,2,3,4,5,6].

A3AR was the last AR to be discovered. Cloning of the cDNA encoding the A3AR homolog from a human heart library was reported over 30 years ago [7]. A3AR is unique in its expression pattern, as under normal physiological conditions, it has low expression levels; however, in cancer and inflammation, its expression level increases in the disease target organ and the peripheral blood mononuclear cells (PBMCs) of the patients [7,8,9]. An example is the development of colon carcinoma from an early stage (polyp) to adenocarcinoma, where it has been shown that the receptor levels increase in a direct correlation to the progression of the disease [10]. An increase in A3AR overexpression is attributed to the upregulation of the transcription factors cyclic AMP response element (CRE)-binding protein (CREB) and nuclear factor kappa B (NF-kB), which are present in the promotor of the A3AR gene, and induce its upregulation [11]. Under these conditions, A3AR over-expression is a manifestation of the disease, rather than its cause.

Synthetic agonists with high affinity and selectivity to A3AR play a dual role under pathological conditions. On one hand, they have a robust anti-cancer and anti-inflammatory effect, leading to specific cell death of tumor and inflammatory cells, while on the other hand, they induce a neuro-, cardio-, and liver-protective effect.

Namodenoson (Cl-IB-MECA; also referred to as CF102, Can-Fite BioPharma, Ltd., Petah Tikva, Israel) is a synthetic ribose-based purine nucleoside with substitutions at the 2, N6, and 5′ positions (2-chloro-N6-(3-iodobenzyl) adenosine-5′-N-methylcarboxamide), leading to A3AR specificity. Namodenoson selectivity for the A3AR is 4750-fold higher than that for A1AR and 1770-fold higher than that for A2AAR (no activity was reported for A2ABR). Its molecular formula is C_18_H_18_CIN_6_O_4_ and its molecular weight is 544.73 Da. As a free base, namodenoson exists as a non-hygroscopic, stable, white-off-white powder. Namodenoson has a Ki of 0.661 nM at the A3AR. It is a very stable agent, hardly metabolized by the liver. This stability allows namodenoson to induce anti-cancer, anti-inflammatory, and protective effects in the liver [11].

In recent years, the role of A3AR in both cancer and inflammation, two conditions that are interrelated and driven by common transcription factors (mainly NF-kB and cytokines), has been extensively investigated. These investigations supported the clinical development of namodenoson as a treatment for metabolic dysfunction-associated steatohepatitis (MASH; formerly known as non-alcoholic steatohepatitis [NASH] [12]) and hepatocellular carcinoma (HCC) [11,13,14,15]. 

This review discusses the potential role of namodenoson in liver diseases by focusing on the evidence related to the duality of the mechanism of action of namodenoson, and how these mechanisms are reflected in preclinical data, as well as in the results of the clinical trials conducted thus far in MASH and HCC. The review also discusses ongoing clinical trials investigating namodenoson for the treatment of these liver diseases.

## 2. Liver Diseases: MASLD, MASH, and HCC

Metabolic dysfunction-associated steatotic liver disease (MASLD, formerly known as non-alcoholic fatty liver disease (NAFLD)), which affects individuals who consume little to no alcohol, is the most common liver disease worldwide, and a leading indication for liver transplantation in developed countries [16,17].

**Figure 1:**
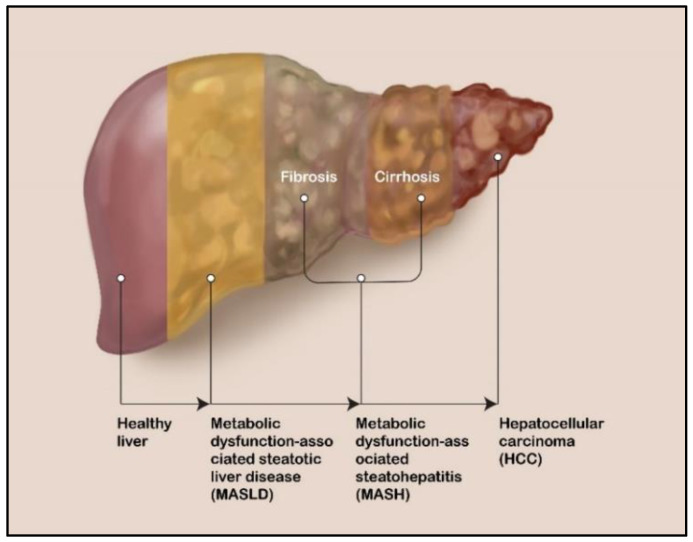
Schematic representation of the development of liver disease from MASLD to HCC.

Recent meta-analyses determined that the global prevalence of MASLD is approximately 30% [18,19]. In its least severe form, MASLD involves steatosis (hepatic fat accumulation). Although most patients with MASLD have no clinically meaningful sequelae, 20–30% of them do progress to its more severe form, MASH, which is characterized not only by steatosis but also by lobular inflammation, hepatocellular swelling, and fibrosis progression. MASH can eventually lead to cirrhosis and HCC (Figure 1) [20,21].

MASLD, MASH, and HCC are all global public health challenges related to the overarching challenges surrounding metabolic syndromes and cancer. The steep rise of the two main risk factors for MASLD, namely obesity and type 2 diabetes mellitus, in recent decades has resulted in a rise in MASLD prevalence [22,23,24]. A recent meta-analysis showed that the global MASLD prevalence increased from 25% in the 1990–2006 timeframe to 38% in the 2016–2019 timeframe, and another meta-analysis demonstrated a similar trend (from 28% in the 2000–2010 timeframe to 32% in the 2011–2021 timeframe) [18,19]. As the prevalence of MASLD increases, so does that of MASH and ultimately that of HCC. A recent analysis utilized a Markov model to estimate the expected MASLD and MASH burdens in 2030. Assuming that the prevalence of obesity and type 2 diabetes will level off in the future, the increase in prevalence of MASLD is expected to be modest, and the total number of MASLD patients by 2030 is expected to be approximately 100 million. In contrast to the modest growth in MASLD cases, the modeling suggests that MASH prevalence will increase by 15–56% by 2030 and that the prevalence of HCC will increase by 47–130% by 2030 due to the aging of the world population [25]. Consequently, MASLD/MASH is the fastest-growing cause of HCC in Western countries [26]. A recent analysis of Medicare patients with HCC involving 13,648 patients suggested that it is now the leading cause of HCC, surpassing viral hepatitis C (MASLD/MASH as the HCC cause: 32% and 20% for inpatients and outpatients, respectively; hepatitis virus C as the HCC cause: 19% and 10%, respectively) [27]. All HCC patients, regardless of the cancer etiologies or risk factors (e.g., MASLD, hepatitis C, hepatitis B, heavy alcohol intake, exposure to environmental toxins such as aflatoxin, obesity, type 2 diabetes, and smoking), constitute 75–85% of all liver cancer cases. In the most recent GLOBOCAN analysis, liver cancer was shown to be the sixth most commonly diagnosed cancer worldwide and the third leading cause of cancer death, with approximately 906,000 new cases and 830,000 deaths in 2020. Interestingly, the analysis also showed that the incidence and mortality rates among men are higher by 2–3 fold than the corresponding rates among women [28].

In patients with MASLD/MASH, HCC can develop with or without existing cirrhosis, although liver cirrhosis is the strongest predictor for HCC development. The annual incidence of HCC in patients with cirrhotic MASH is up to 12% [29]. HCC develops in these patients due to the combination of insulin resistance, damage to the mitochondria from the accumulation of fat, inflammation, and chronic dysregulation of cytokines [29]. Despite advances in HCC therapies, including the availability of immune checkpoint inhibitors, further research is greatly needed, as the overall survival with the current systemic therapies is limited (average 5-year survival as low as 2.5% for advanced metastatic disease) [30,31].

Thus, clearly, effective approaches for treating MASLD/MASH and preventing its progression to cirrhosis and ultimately to HCC are urgently needed, as are effective treatments for HCC.

## 3. Namodenoson Molecular Mechanism of Action

Namodenoson induces a differential effect on pathological and normal body cells. A3AR is highly expressed on the cell surface of inflamed and cancer cells, whereas normal cells have low A3AR expression. The density of the receptor on the cell surface determines its response to namodenoson.

### 3.1. The Effect of Namodenoson on Liver Inflammatory and Cancer Cells

**Figure 2:**
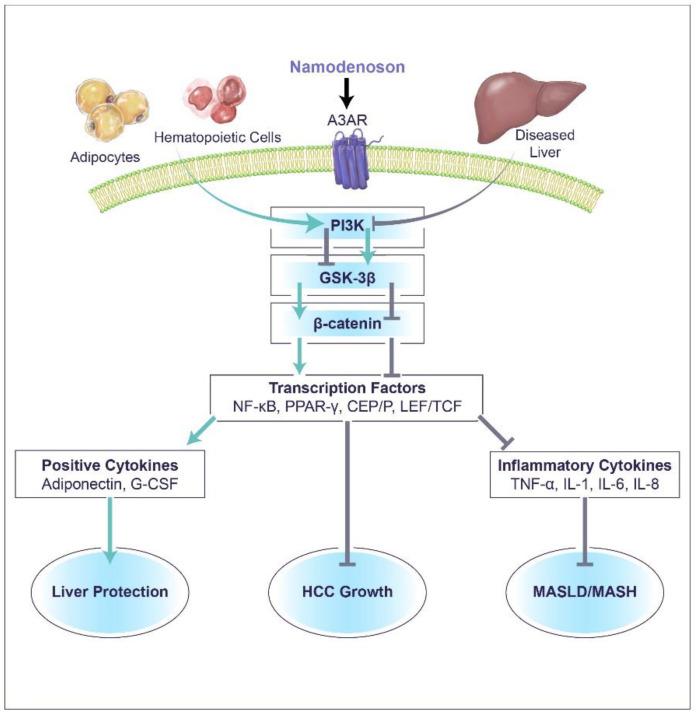
Schematic representation of the multifaceted mechanism-of-action of namodenoson. The scheme demonstrates the effect of namodenoson on diseased liver, hematopoietic cells, and adipocytes through its binding to A3AR. This binding leads to deregulation of PI3K, which ultimately results in inhibition of HCC growth, inhibition of MASLD/MASH-associated inflammation, as well as to liver protection. Grey lines represent inhibitory effects; green arrows represent activation effects.

Cancer and inflammatory liver cells respond to namodenoson through the same molecular mechanism (Figure 2). In vitro, human stellate cells, N1S1 cells, and HEP-3b HCC cells were used, whereas in vivo, experimental models including concanavalin A (Con A)-induced liver inflammation, carbon tetrachloride (CCL4), and STAM MASLD/MASH models, as well as N1S1 and HEP-3b HCC murine models were employed [9,32,33,34,35]. Upon treatment with namodenoson, a decrease in cAMP and phosphoinositide 3-kinase (PI3K) was noted [9,33]. PI3K is a key therapeutic target for cancer and inflammation based on findings showing that over-expression of PI3K is significantly correlated with human tumor progression and deterioration of inflammation [36,37].

PI3K inhibition induces modulation of signal transduction pathways in liver inflammation and HCC. Specifically, a decrease in the expression level of IKB, IKK, and the transcription factor NF-κB and the subsequent decrease in tumor necrosis factor alpha (TNF-α), which directly acts as an anti-proliferative factor in HCC cells, was reported [33,34,35].

Through the PI3K inhibition, namodenoson induces GSK-3β upregulation, a key component of the Wnt signaling pathway, known to phosphorylate β-catenin and to induce its ubiquitination, thereby preventing its association with LEF/TCF and the translocation of the complex to the nucleus. As a result, a decrease in additional transcription factors, including CEB/P, LEF/TCF, and PPAR-γ, occurs, leading to inhibition of inflammatory cytokines such as TNF-α, interleukin (IL)-1, IL-6, IL-8, and more [38,39,40]. Moreover, the decrease in β-catenin prompts upregulation of the pro-apoptotic proteins BAD, BAX, Bcl2, and caspase-3, leading to inhibition of HCC cells via apoptosis [9].

### 3.2. The Effect of Namodenoson on Normal Cells

Namodenoson affects normal cells in an opposite way to its effect on cancer and inflamed cells through an induction of upregulation of cAMP and PI3K (Figure 2). This effect leads to the production of positive cytokines [34,41,42]. The affected cytokines include granulocyte-colony stimulating factor (G-CSF) and adiponectin. Hematopoietic cells, and specifically bone marrow cells, treated with namodenoson were shown to induce G-CSF production through upregulation of IKK and NF-kB, the transcription factor associated with G-CSF [41]. The main action of G-CSF is stimulation of the production of neutrophils, as well as their mobilization, survival, and chemotaxis [43]. In oncology clinical practice, G-CSF is typically used to decrease the severity and duration of chemotherapy-induced neutropenia [44,45]. In recent years, G-CSF has been introduced as a treatment for liver diseases such as severe alcoholic hepatitis, decompensated liver cirrhosis, and acute-on-chronic liver failure. Clinical studies demonstrated that G-CSF can mobilize hematopoietic stem cells (CD34 cells) and thus improve liver function, potentially facilitating its recovery from injury. It has also been suggested that treatment with G-CSF leads to fewer infectious complications as well as improved survival in these patient populations [46,47,48].

Namodenoson also binds to A3AR on adipocytes, leading to upregulation of adiponectin, an adipocyte-derived cytokine that is an abundant serum protein [15,34,49]. Adiponectin has a protective role in the regulation of metabolism, inflammation, and cancer. The levels of adiponectin decrease in various pathological states including insulin resistance, obesity, metabolic syndrome, and cardiovascular diseases [50]. In a STAM model where streptozotocin-injected mice were fed a high-fat diet and then treated with namodenoson, elevation in adiponectin levels were noted alongside improvement in the clinical signs of liver inflammation and fibrosis [34]. These preclinical findings were consistent with results from the phase 2B study of namodenoson in MASLD/MASH, where increased levels of adiponectin in the serum of patients treated with namodenoson were recorded [15]. Moreover, these findings are also in line with other studies showing that increased adiponectin levels are associated with marked improvement in liver diseases such as alcoholic liver disease (ALD), hepatic fibrosis, MASLD/MASH, and HCC [51,52,53,54]. Adiponectin levels in the plasma are lower in obese individuals vs. those with normal weight, and obesity has been associated in epidemiologic studies with many common cancers, with the strongest evidence for digestive system cancers, including liver cancer [55]. In vitro and in vivo evidence also support the association of lower levels of adiponectin with tumor-promoting pathways and higher levels of adiponectin with inhibition of processes such as cell proliferation, migration, and invasion [53]. For example, adiponectin treatment has been shown to inhibit growth of HCC cells (HepG2, Huh7) in a dose-dependent manner; however, this effect was not observed when a normal human hepatocyte cell line (THLE-2) was treated with adiponectin [56]. Adiponectin, expressed via a recombinant adenovirus, also inhibited HCC growth in nude mouse models, compared to control (saline or adenovirus-luciferase) [56].

In addition, adiponectin was also shown to display regenerative properties in the liver [57,58]. In a preclinical study, regeneration of the liver after partial hepatectomy in knockout mice deficient of adiponectin (Adn^−/−^) was delayed compared to wild-type mice. However, cell cycle progression was accelerated in the knockout mice relative to the wild-type mice, suggesting that adiponectin has multiple effects in the liver. This dynamic modulation effect was further supported by the observation that Adn knockout mice reduced the response of hepatocytes to IL-6 and increased bioavailability of growth factors [57].

## 4. Namodenoson for the Treatment of MASLD/MASH

### 4.1. Preclinical Evidence

The anti-MASLD/MASH effect of namodenoson has been demonstrated in two animal models of the disease [34]. In a STAM model where mice were fed a high-fat diet and MASH was induced by subcutaneous administration of streptozotocin, oral administration of namodenoson, compared to vehicle, led to significant reductions in steatosis, and improvements in both hepatocyte ballooning and lobular inflammation. Evaluating the disease activity score (NAFLD-activity score (NAS)), which is a composite score combining the three aforementioned elements according to the Kleiner criteria [59], demonstrated a significant decrease in the NAS score in the namodenoson-treated group vs. the vehicle [34]. In another murine model where liver fibrosis was induced by intraperitoneal CCL4 injection, the significant increase in serum alanine transaminase (ALT) observed in the mice after the CCL4 injection was reversed by intraperitoneal injection of namodenoson but not the vehicle. Treatment with namodenoson also significantly reversed the CCL4-induced ascites (compared to the vehicle). Liver sections derived from the CCL4-treated mice showed increased inflammation and fibrosis, which were both reduced significantly (compared to vehicle) upon treatment with namodenoson [34]. In another preclinical study, namodenoson had a positive effect in rat models of liver ischemia/reperfusion (induced by clamping the hepatic vasculature for 30 min) and partial hepatectomy (70% of the liver). This observed effect may be due to an anti-inflammatory and anti-apoptotic effect in the liver, which further supports the hepatoprotective characteristics of namodenoson and its potential clinical utility in MASLD/MASH [60].

### 4.2. Clinical Evidence

The preclinical evidence regarding the hepatoprotective effects of namodenoson prompted its clinical development as a treatment for MASLD/MASH. A phase 2 study in patients with MASLD/MASH was conducted [15]. The details of this phase 2 study are summarized in Table 1. This was a randomized (1:1:1) double-blind study in 60 patients with MASLD (ALT ≥ 60 IU/L), which compared two doses of namodenoson (12.5 mg BID and 25 mg BID for 12 weeks, follow up was for 16 weeks) to placebo. The main objective of the study was to investigate the anti-inflammatory effect of namodenoson by examining the levels of serum ALT and aspartate transaminase (AST) over time. A dose-dependent decrease in serum ALT levels over time in the namodenoson arm was observed. This decrease trended towards significance at 12 weeks for the 25 mg BID dose (change from baseline [CFB] for ALT vs. placebo, *p* = 0.066). Normalization of ALT levels at Week 12 was reported for 31.6% of patients in the namodenoson 25 mg BID arm vs. 20.0% of patients in the placebo arm (*p* = 0.405). At Week 16, ALT normalization was reported for 36.8% and 10.0% of patients in the namodenoson and placebo arms, respectively (*p* = 0.038). A dose-dependent decrease in serum AST levels was also observed (CFB for 25 mg BID vs. placebo at Week 12, *p* = 0.03). In addition, increased adiponectin levels were noted between baseline and Week 12 (mean CFB for the 12.5 mg BID vs. placebo, 539 ng/mL vs. −78 ng/mL, *p* = 0.032), further supporting the anti-inflammatory effect of namodenoson [15].

A secondary objective of this study was to determine the impact of namodenoson on the liver fat content and fibrosis progression. The study demonstrated a decrease in liver fat volume at Week 12 (mean CFB −158.0 mL; *p* = 0.065 vs. placebo). Furthermore, the proportion of patients with high steatosis scores (controlled attenuation parameter (CAP) score ≥ 331) decreased in both the namodenoson arms at Week 12 compared to screening (12.5 mg BID: 50% to 31%; 25 mg BID: 43% to 14%) and increased in the placebo arm (from 33% to 40%; differences between the treatment arms and placebo were not statistically significant). Namodenoson treatment also led to a decrease in Fib4-scores (a non-invasive index for liver fibrosis based on AST, ALT, platelet count and age) from screening to Week 12 (Week 12 mean CFB, −0.08, −0.28, and −0.04 for the 12.5 mg BID, 25 mg BID, and placebo, respectively; *p* = 0.011 for 25 mg BID vs. placebo). Furthermore, the effect on liver fibrosis was also demonstrated by the decreased proportion of patient with MASH (as defined by FibroScan-AST^®^ (FAST) score, a combination of FibroScan^®^-determined CAP, liver stiffness measurement (LSM), and AST, >0.67) from screening to Week 12 in both namodenoson arms (within group comparison Week 12 vs. screening: 12.5 mg BID, *p* = 0.077; 25 mg BID, *p* = 0.002) [15].

In addition, a linear decrease in body weight was observed in all study arms during the study with a greatest decrease in the namodenoson 25 mg BID arm (Week 16, a mean loss of 2.1 (SE, 0.7) kg) followed by the 12.5 mg BID arm (mean, 1.6 (SE, 0.7) kg) and the placebo arm (mean, 0.5 (SE, 0.7) kg). The CFB difference between the namodenoson arms and placebo were not statistically significant [15].

Treatment with either dose of namodenoson was well tolerated. No drug-emergent severe adverse events (AEs), drug-related withdrawals, hepatotoxicity, or deaths were reported [15].

The efficacy of namodenoson, and particularly the 25 mg BID dose coupled with the observed safety prompted the design of the currently recruiting randomized double-blind placebo-controlled phase 2B trial (ClinicalTrials.gov identifier: NCT 04697810). In this study, 114 patients with biopsy-proven MASH will be randomly assigned (in a 2:1 ratio) to namodenoson 25 mg BID or placebo for 36 weeks, at which time they will undergo post-treatment liver biopsy to determine their NAS score. The primary endpoints of this trial include the proportion of patients with ≥2 point improvement in NAS and safety [61].

## 5. Namodenoson for the Treatment of HCC

### 5.1. Preclinical Evidence

Analysis of mRNA A3AR expression in tumor lesions and adjacent normal tissues from patients with HCC (*n* = 21, of whom 61% also suffered from cirrhosis) demonstrated increased A3AR expression levels in tumors but not in the surrounding normal tissues. Interestingly, the high expression level was also reflected in the PBMCs of these patients, whereas in PBMCs of healthy volunteers, the A3AR expression was low. Interestingly, PBMCs from patients who also had cirrhosis had an increase of 48% in the expression levels of A3AR compared to HCC patients without cirrhosis [9].

Furthermore, A3AR was overexpressed in tumor tissues derived from HCC tumor-bearing rats vs. normal liver tissue from naïve rats, and in PBMCs from HCC tumor-bearing rats vs. naïve rats [9]. The anti-cancer effect of namodenoson (administered orally) was then demonstrated in a preclinical rat orthotopic model. Namodenoson demonstrated a remarkable bell-shaped dose-dependent inhibitory effect on tumor growth in the liver in rats that were injected with the N1S1 rat cell line in the right hepatic lobe. The maximal effect was at a dose of 100 μg/kg (92.8 ± 6.9% inhibition). In addition to the reduction in size, histological images of liver sections derived from namodenoson-treated rats demonstrated several irregular areas of necroapoptosis [9].

### 5.2. Clinical Evidence

Following the preclinical evidence, two clinical studies in patients with advanced HCC were conducted. These studies are summarized in Table 1. The first was a phase 1/2 open-label dose-escalation study involving 18 patients with advanced, unresectable HCC (6 at each dose level: 1, 5, and 25 mg BID) [13]. Twelve of these patients failed prior sorafenib treatment, thirteen were categorized as having Child-Pugh class A (CPA), and five as having Child–Pugh class B (CPB) hepatic dysfunction. No dose-limiting toxicities or serious drug-related AEs were reported. Preliminary evidence of antitumor activity and pharmacokinetic effects were observed. The median overall survival (OS) for the entire cohort was 7.8 months, and for CPB patients, 8.1 months. Stable disease by Response Evaluation Criteria in Solid Tumors (RECIST) for at least four months was reported in four patients. Pharmacokinetic analysis revealed a correlation between A3AR overexpression at baseline and patients’ overall survival [13].

Due to the efficacy signal observed in advanced HCC patients with CPB, the phase 2 study focused on this population, and specifically on HCC CPB patients who previously received sorafenib and either progressed on it or could not tolerate it [14]. The 78 patients included in this study were randomized (2:1) to namodenoson 25 mg BID (*n* = 50) or placebo (*n* = 28) until discontinuation due to intolerance, withdrawal of consent, or death. Patients who continued blinded treatment were offered open label namodenoson (25 mg BID) upon unblinding. While the study did not achieve its primary endpoint, which was OS (median OS was 4.1 and 4.3 months for namodenoson and placebo, respectively; hazard ratio (HR), 0.82; 95% confidence interval (CI) 0.49–1.38; *p* = 0.46)), it did achieve superiority in survival in the largest subpopulation of patients with CPB disease and a Child–Pugh score of 7. This subgroup included 56 patients (34 in the namodenoson arm and 22 in the placebo arm). In this subgroup, the difference between the namodenoson and placebo arms in 12-month OS was statistically significant (44% vs. 18%, respectively; *p* = 0.028). Moreover, nonsignificant improvements in OS and progression-free survival (PFS) with namodenoson were also noted in this subgroup. The median OS was 6.9 vs. 4.3 months (HR, 0.81; 95% CI: 0.45–1.43, *p* = 0.46), and the median PFS was 3.5 vs. 1.9 months (HR, 0.89; 95% CI: 0.51–1.55, *p* = 0.67). Among all patients eligible for response evaluation (i.e., patients with at least one post-baseline assessment; 34 in the namodenoson arm and 21 in the placebo arm), partial response (RECIST criteria) was reported for 9% in the namodenoson arm group vs. 0% in the placebo arm [14].

The safety profile of namodenoson in the study was excellent and consistent with that observed in the phase 1/2 study. No treatment-related deaths were reported, and no patients withdrew due to toxicity [14].

Interestingly, one patient (female, aged 61 years at enrollment) who was randomized to the namodenoson arm and continued treatment with namodenoson for more than six years under the open-label extension, demonstrated partial response after approximately seven weeks. However, within four years of namodenoson treatment, this patient experienced a complete response, as manifested by the disappearance of the tumor mass, ascites, and peritoneal carcinomatosis (observed in computed tomography scans), alongside normalization of serum ALT and AST levels. Her treatment is ongoing [62].

The findings of the phase 2 trial support the continued clinical development of namodenoson as second-line treatment for patients with HCC CPB and a Child–Pugh score of 7. A randomized double-blind, placebo-controlled phase 3 study (LIVERATION) in this population is currently recruiting patients (ClinicalTrials.gov identifier: NCT05201404). In this study, 417 patients will be randomly assigned (2:1 ratio) to namodenoson (25 mg BID) administered until disease progression or unacceptable tolerability or placebo. The primary endpoint is OS with a planned follow up of five years [63]. Interim analysis is also planned.

Namodenoson received an Orphan Drug status by both the Food and Drug Administration (FDA) and the European Medicines Agency (EMA), as well as Fast Track Status by the FDA [64].

## 6. Conclusions

Namodenoson’s multifaceted mechanism of action involves its binding to A3AR on inflammatory and cancer cells in the diseased liver as well as to A3AR on bone marrow cells and adipocytes. In HCC, it leads to deregulation of the Wnt/β-catenin and NF-κB pathways and inhibition of multiple transcription factors including NF-κB, ultimately inhibition of HCC growth via apoptosis. In MASLD/MASH, binding to the A3AR on adipocytes and bone marrow cells leads to stimulation of transcription factors, including NF-κB, resulting in up-regulation of G-CSF and adiponectin and hepatoprotective effects. This unique mechanism-of-action is consistent with the observed effects of namodenoson as a non-cytotoxic anti-inflammatory and anti-cancer agent in the liver, as well as with its liver protective effects in clinical studies. The observed efficacy of namodenoson, alongside its favorable safety profile, positions it as a promising drug candidate for the treatment of two liver diseases, MASLD/MASH and HCC. Furthermore, since MASLD/MASH can develop over time into HCC, namodenoson has the potential to treat MASLD/MASH and prevent its deterioration into HCC. The clinical development of namodenoson as a treatment for both diseases is currently ongoing with results expected in the coming years.

## Figures and Tables

**Table 1:** A summary of clinical studies investigating namodenoson in MASLD/MASH and HCC.

Author Reference	Disease	Phase, Study Design, *n*, Key Endpoints	Key Findings
Safadi et al. [15]	MASLD with or without MASH	Phase 2, randomized (1:1:1) double blind study of namodenoson 12.5 mg BID (*n* = 21) or 25 mg BID (*n* = 19) vs. placebo (*n* = 20). Main endpoints: ALT after 12 weeks, safety.	Change from baseline in serum ALT levels over time: A trend towards significance at Week 12 for namodenoson 25 mg BID vs. placebo (*p* = 0.066). ALT normalization rate: A statistically significant difference at Week 16, for 25 mg BID vs. placebo (*p* = 0.038). Safety: Namodenoson was well tolerated
Stemmer et al. [13]	Advanced unresectable HCC	Phase 1/2 open-label dose-escalation study (*n* = 18, 6 at each dose level: 1, 5, and 25 mg BID). Main endpoint: Safety	Safety: Namodenoson was not associated with dose-limiting toxicities or serious drug-related AEs.
Stemmer et al. [14]	HCC CPB patients who either progressed on, or could not tolerate, prior sorafenib treatment.	Phase 2, randomized (2:1) double blind study of namodenoson 25 mg BID (*n* = 50) vs. placebo (*n* = 28). Main endpoint: OS in the ITT population.	OS—ITT: Primary endpoint was not met. Median OS, 4.1 and 4.3 months for namodenoson and placebo, respectively; HR, 0.82; 95% CI, 0.49–1.38; *p* = 0.46. 12-month OS in patients with Child Pugh score of 7 (*n* = 56): 44% and 18%, for namodenoson and placebo, respectively; *p* = 0.028. Safety: Namodenoson was well tolerated

AE, adverse event; ALT, alanine transaminase; CI, confidence interval; CPB, Child-Pugh class B; HCC, hepatocellular carcinoma; HR, hazard ratio; ITT, intention-to-treat; MASH, metabolic dysfunction-associated steatohepatitis; MASLD, metabolic dysfunction-associated steatohepatitis; OS, overall survival.
